# Pain neuroscience education and physical exercise for patients with chronic spinal pain in primary healthcare: a randomised trial protocol

**DOI:** 10.1186/s12891-019-2889-1

**Published:** 2019-11-03

**Authors:** Miguel A. Galán-Martín, Federico Montero-Cuadrado, Enrique Lluch-Girbes, M. Carmen Coca-López, Agustín Mayo-Iscar, Antonio Cuesta-Vargas

**Affiliations:** 1 Unit for Active Coping Strategies for Pain in Primary Care, East-Valladolid Primary Care Management, Castilla and Leon Public Health System (Sacyl), Valladolid, Spain; 2 Doctoral Program of Research in Health Sciences, University of Valladolid, Valladolid, Spain; 3 Department of Physical Therapy, University of Valencia, Valencia, Spain; 4 “Pain in motion” International Research Group, Brussels, Belgium; 5 Department of Human Physiology and Rehabilitation Sciences, Faculty of Physiotherapy, Vrije university Brussels, Brussels, Belgium; 6 Castilla and Leon Regional Centre of Sports Medicine, Castilla and Leon Public Health System (Sacyl), Valladolid, Spain; 7 Department of Statistics and Operational Research and IMUVA, University of Valladolid, Valladolid, Spain; 8 Department of Physiotherapy, Faculty of Heath Sciences, University of Malaga, Málaga, Spain; 9 Institute of Biomedical Research in Malaga. IBIMA, Málaga, Spain; 10 School of Clinical Science, Faculty of Health Science, Queensland University Technology, Brisbane, Australia

**Keywords:** Chronic pain, Chronic spinal pain, Pain neuroscience education, Physical exercise, Treatment protocol, Primary care

## Abstract

**Background:**

Chronic musculoskeletal pain affects more than 20% of the population, and the prevalence is increasing, causing suffering, loss of quality of life, disability, and an enormous expenditure on healthcare resources. The most common location for chronic pain is the spine. Many of the treatments used are mainly passive (pharmacological and invasive) and poor outcomes. The treatments currently applied in the public health system do not comply with the recommendations of the main clinical practice guidelines, which suggest the use of educational measures and physical exercise as the first-line treatment. A protocol based on active coping strategies is described, which will be evaluated through a clinical trial and which could facilitate the transfer of the recommendations of the clinical practice guidelines to a primary care setting.

**Methods:**

Randomised and multicentre clinical trials, which will be carried out in 10 Primary Care centres. The trial will compare the effect of a Pain Neuroscience Education program (six sessions, 10 h) and group physical exercise (18 sessions program carried out in six weeks, 18 h), with usual care physiotherapy treatment. Group physical exercise incorporates dual tasks, gaming, and reinforcement of contents of the educational program. The aim is to assess the effect of the intervention on quality of life, as well as on pain, disability, catastrophism, kinesiophobia, central sensitisation, and drug use. The outcome variables will be measured at the beginning of the intervention, after the intervention (week 11), at six months, and a year.

**Discussion:**

Therapeutic interventions based on active coping strategies are essential for the treatment of chronic pain and the sustainability of the Public Health System. Demonstrating whether group interventions have an effect size is essential for optimising resources in such a prevalent problem.

**Trial registration:**

NCT03654235 “Retrospectively registered” 31 August 2018.

## Background

Chronic musculoskeletal pain (CMP) affects about 20% of the population in western countries [1–3], causing suffering, disability, and a significant loss of quality of life in patients affected by it [3, 4]. It also has a great economic impact related to lost working hours and consumption of health resources [2, 5]. In Primary Care (PC), CMP is one of the most common reasons for consultation, being chronic spinal pain (CSP) the most frequent, and with a growing prevalence [6, 7].

PC has not been giving a satisfactory response to the needs of these patients. General practitioners working in PC devote much of their regular workday to seeing patients with CSP, without being quite certain about what the best therapeutic option is in these cases (Pharmacological and non-pharmacological) [8]. The possibilities for referral are multiple, with no certainty that one option is better than the other such as referral to PC physiotherapy, or referral to different specialised care units (traumatology, rheumatology, neurosurgery, the pain care unit, etc.). Even so, the majority of treatments administered to these patients are focused on the treatment of structures that are considered pain generators (muscles, joints, and stenosis), and in blocking the nociceptive pathways, in both cases with poor results [9]. Therefore, a new therapeutic approach is necessary for the treatment of these patients, and in this sense, advances in neuroscience that have occurred in the past years allow us to know that in CSP, central pain processing mechanisms are involved in the maintenance and perpetuation of the painful experience [10, 11].

The clinical characteristics of Central Sensitisation (CS) presented by the majority of these patients have been meticulously detailed [10, 12, 13]. In addition, structural changes have been reported at the cerebral level, with decreased density and volume of grey matter in prefrontal and dorsolateral cortex, thalamus, brainstem, and somatosensory cortex [14–16], as well as an increase in grey matter volume in the right hippocampus and parahippocampal gyrus [17]. These changes that appear when pain is perpetuated [18], seem to be involved in the dysfunction of the descending inhibitory pathways [10, 19], motor control alterations [20, 21], and the emergence of neural phenomena of long-term potentiation [22] that these patients present. In addition, it is known that patients with CS present kinesiophobia [23], fear-avoidance behaviours [24], disability [2, 25], and high catastrophism levels [26]. Based on this new knowledge, therapeutic approaches are required to reverse or improve the changes described, and in this sense, the most recent clinical practice guidelines recommend patient education and physical exercise in CSP as the first line of action [27–31].

Therefore, a therapeutic approach is necessary to improve functionality, as well as tools to reverse the structural changes that present in some cerebral areas in patients with CSP and CS. Within the educational approach recommended by the guidelines, pain neuroscience education (PNE) as a health education strategy allows changing cognitions by modifying erroneous beliefs and reduces catastrophism, kinesiophobia, and fear-avoidance behaviours [32].

It has been verified that PNE interventions improve their outcomes if they are combined with a physical exercise (PE) directed program [33]. PE is a first-line recommendation in clinical practice guidelines [27–31], and in this sense, aerobic PE has proven to be a great ally in the treatment of CSP [34, 35].

It is also known that certain exercises facilitate neurogenesis, produce neuroplastic changes at the cerebral level [36–39] and activate descending pain inhibitory pathways [40]. In addition, PE improves the functional status of these patients by improving motor control, achieving reduced disability. Therefore, a therapeutic approach based on PNE and a PE directed program, could be a useful tool to improve the quality of life, disability, and to achieve a decrease in perceived pain intensity. With this model that combines PNE and PE, several clinical trials and reviews have been carried out with hopeful outcomes [32, 41–43], but there is great variability in terms of the time dedicated to educational intervention, therapeutic PE program, and the way to perform this exercise program. Most studies on PNE and PE have been carried out in Anglo–Saxon and northern European countries. Taking into account that psychosocial aspects are important in the chronification of pain, and since distinct cultural environments may have a different influence on these psychosocial aspects [44], it is necessary to develop a protocol adapted to our environment (Spain and similar cultures of southern Europe), and conduct a clinical trial to assess the intervention effect. Trials of this kind have not been made yet in PC in our country.

For the reasons shown, a program has been designed based on active coping strategies that include PNE and PE, and that program has been adapted to our healthcare context of PC. The protocol proposed for the evaluation would allow transferring the clinical practice guidelines recommendations to the field of PC, since it has been detected that the clinicians in PC do not have a clear certainty on how to educate CSP patients, and what the best type of PE that can be prescribed. The designed program, which was born and developed in the National Health Service (SNS), in the Castilla y León division (SACYL), aims to optimise the available resources in PC and in order to have a greater reach, we have opted in its design a group intervention which will be described later on. Group interventions have proven to have high therapeutic potential [45–47], and do not exclude that, at the individual level, each patient with CSP can receive specific instructions. In the second part of the program, playful (gamification) and double task components are added to the PE program, aimed at activating new neural circuits which promote neurogenesis and neuroplasticity. In addition, the proposed therapeutic approach encourages the acquisition of healthy lifestyles that can have a beneficial effect on comorbidities, present or potential, in CSP patients (Obesity, HTA, type 2 diabetes, risk of fragility fractures, etc.).

The proposed treatment aims to change cognition and erroneous beliefs in patients with CSP, as well as improve functionality and physical condition. The main objective of the study is to improve the quality of life of patients with CSP. As secondary objectives, the aim is to reduce the levels of catastrophism, kinesiophobia, central sensitisation, disability, and pain intensity. It is also intended to register if the proposed approach decreases the drug consumption and the frequency of the health services for CSP patients. If the results of the intervention are favourable, it is intended, in a second phase of the study, to check for structural and functional changes in brain structures of CSP patients, by using Functional Magnetic Resonance imaging (fMRI) and Diffusion Magnetic Resonance imaging (dMRI).

Therefore, it is proposed to evaluate the effectiveness and cost-effectiveness of a programme based on active coping strategies that include PNE and PE by means of a randomised clinical trial compared to usual care in PC physiotherapy. The protocol outlined describes the characteristics of an educational and physical exercise program, which could be implemented in PC centres of the Spanish National Health Service in case the results are favourable.

## Methods and study design

The clinical trial has been registered (Clinicaltrials.gov NCT03654235) and has received approval from the Ethics Committee of Clinical Investigation (CEIC) of the East Valladolid health area (CASVE-NM _ 16–252) and of the CEIC of the West Valladolid health area (CEIC: 26/17).

This study protocol describes the design of a multicentric randomised clinical trial (RCT) of parallel groups (ratio 1:1) to be carried out in 10 health centres within the Public Health network areas of East Valladolid and West Valladolid, belonging to the public health service of Castilla y León (Spain). The study protocol conforms with the standard protocol items: Recommendations for Interventional Trials (SPIRIT) [48] while the RCT conforms to the Consolidated Standards of Reporting Trials (CONSORT) [49].

Patients will be recruited from family medicine consultations and will be referred to PC physiotherapy units. For patient recruitment, the questionnaire contained in Annex I are used. The RCT flow diagram is shown in Annex I.

### Inclusion criteria

The patients will be recruited from the PC medical consultations in accordance with the following inclusion criteria:

- Patients of both sexes between 18 and 70 years old.
- Non-specific back pain of at least six months (The presence of pain in other regions, in addition to the back, will not be grounds for exclusion)
- Agree to participate in the study and sign informed consent.

### Exclusion criteria

- Oncological pain.
- Spine fracture or surgical intervention in the previous year.
- The neurological cognitive alteration that prevents understanding the contents of the PNE program (In case of doubt, assessment with the Minimental test will be conducted [50]. The minimum score is 25 [51])
- Physical performance deficit that prevents the execution of the planned PE program (Minimum requirement: execution in normal time (10″) of the Timed Up and Go test).
- Pregnancy.
- Cauda equina syndrome.
- Patients presenting other clinical conditions that may aggravate chronic spinal pain (chronic fatigue syndrome, fibromyalgia and complex regional pain syndrome).
- Patients with associated pathologies that make it impossible to perform a physical exercise program (myopathies, neurological diseases, etc., with significant impairment of functionality)
- Patients on treatment with alternative therapies.

### Recruitment method

Patients attending family medicine and physiotherapy consultations at the participating centres that meet the inclusion criteria will be invited to participate in the RCT. Physicians and physiotherapists involved in the recruitment process will receive a clinical session to ensure the selection process is carried out in compliance with the inclusion criteria, and that the participants do not meet the exclusion criteria. Patients will receive oral information and a written document (Patient information sheet). If the patient is interested in participating in the study, after signing the informed consent, he will be cited to be evaluated by an external evaluator who will perform the initial assessment. In the same act of the informed consent, the patient will receive a revocation sheet in case they decide to leave the study at any time. After the initial evaluation, participants will be randomly assigned to one of the two study groups (experimental or control).

### Randomisation

After the initial evaluation, each patient will be assigned an alphanumeric code. The alphabetic part of the code will correspond to the participant’s health centre, while the numerical part is allocated by correlative numbers. To perform the randomisation procedure, the Health Technician of the health area of East Valladolid will receive a list of the alphanumeric codes of the participants and make a random allocation using the SPSS statistical package.

### Blinding

All evaluations will be performed by evaluators who do not know the group to which the patient has been assigned. An independent biostatistician will perform the statistical analysis without knowing the treatment carried out by each group. It will not be possible to blind the intervention performed or the physiotherapists who perform it, but physiotherapists who perform the intervention do not participate in the patient evaluation process.

### Interventions

#### A brief descrption of trial's arms are shown in Table 1. The intervention of the experimental group

**Fig. 1:**
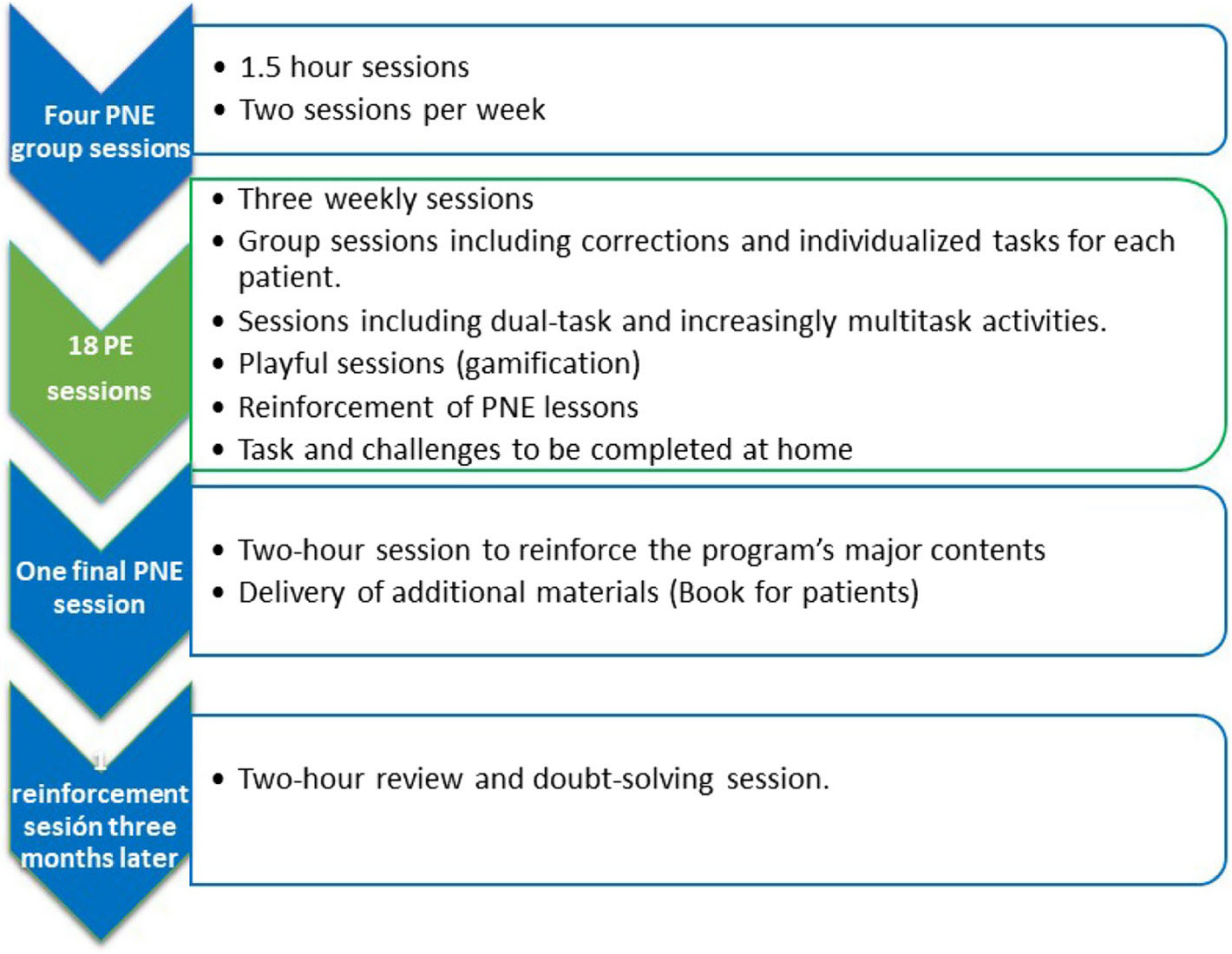
Temporal distribution of program sessions (experimental group)

Patients assigned to the experimental group will perform a PNE program consisting of six sessions (10 h) and 18 sessions of therapeutic PE to be performed in six weeks (18 h), with a frequency of three sessions per week. The program will be carried out according to the order shown in Fig. 1.

**Table 1:** A brief description of the trial’s arms

Arms	Assigned Interventions
*Experimental: PNE and PE program* • *Pain neuroscience education (Health education)* • *Physical exercise program*	*Health Education* *Six pain neuroscience education sessions (10 h) and delivery of printed reinforcement material.* *Physical exercise* *Group physical exercise program (18 sessions: three sessions/week) led by a physiotherapist. It includes exercises to improve strength, coordination, balance, and aerobic capacity. Work with double tasks, recreational activities to overcome kinesiophobia and activities to do at home are used in the program.*
*Active Comparator: Usual care in Primary Care Physiotherapy* • *Usual care in Primary Care Physiotherapy Units*	*Usual care in Primary Care Physiotherapy Units* *Treatment supported by the primary care physiotherapy protocol in the health service of Castilla y León that was in force at the time of the intervention. Patients receive 15 sessions of analgesic electrotherapy, thermotherapy and standardised physical exercise.*

PNE is a health education intervention that aims to provide up-to-date information on neuroscience advances in the field of chronic pain. For this purpose, a group educational program has been designed, the contents of which appear in the Additional file 1. Pedagogical resources that facilitate the learning process will be used to enhance knowledge transfer. The times in which patients are merely information receivers, and the times in which they actively participate, will be adequately dosed. Care has been taken with the graphic material that will accompany the presentations, and infographics have been created to facilitate the process. All sessions will use explanatory and motivational videos. The educational intervention will be essential to raise awareness of the need for lifestyle changes and ensure that participants arrive at the second part of the program, which will start the PE, in an “Action Stage”, following the transtheoretical model of Prochaska and Diclemente [52, 53].

PE is carried out in group form, although this will not be an impediment for each participant during the session to receive individualised advice from the physiotherapist. During the sessions, references to the theoretical contents that have been learned in the first part of the program will be made repeatedly. It is essential that patients understand what they do and why they do it. Since patients with CSP have an alteration in their pain inhibitory system [54], the dosage of the scheduled exercise requires taking certain precautions and adapting the exercise dose to the patient’s functional status [34]. The inhibitory system dysfunction may cause an increase in pain during the first PE sessions. Aware of this fact, it will be necessary to be prepared so that this transient increase in symptoms is not lived with alarmism. The appearance of pain during exercise, as long as we move at appropriate levels of intensity, will never be a reason to stop the activity [55, 56]. At all times, during the PE sessions, the increase in functionality is sought as a reference. This aspect will be previously reinforced in the education sessions.

**Fig. 2:**
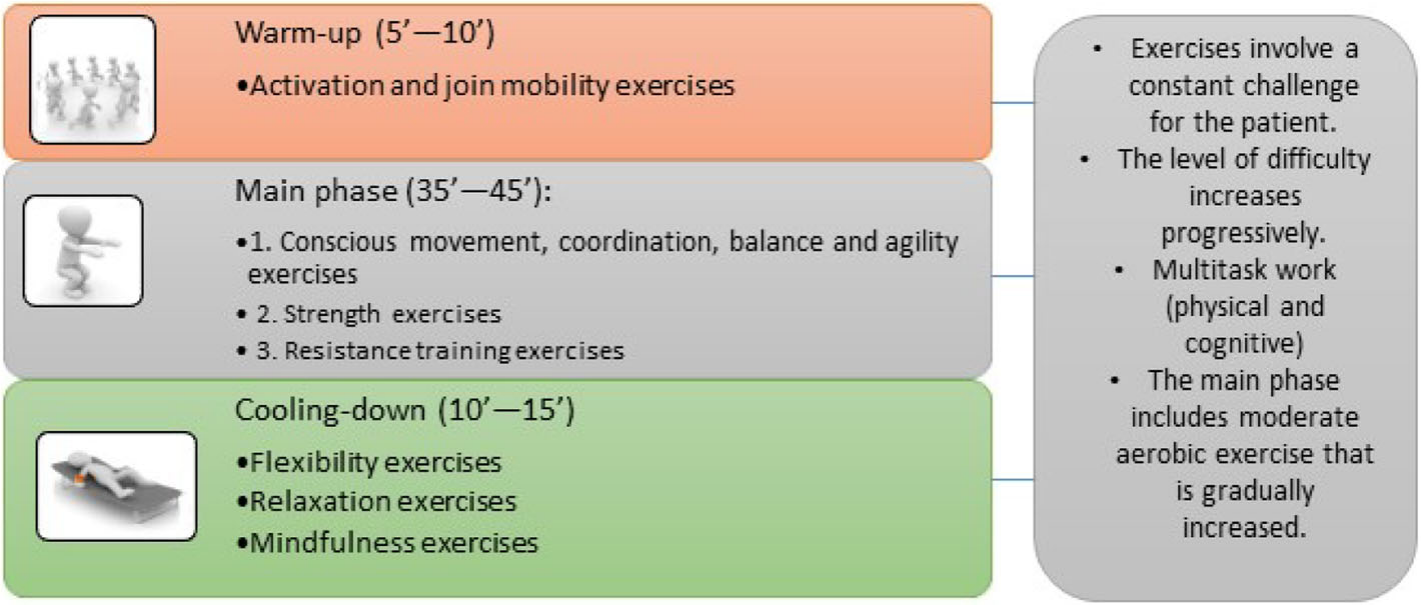
Structure of sessions (experimental group)

PE sessions will have a basic structure that complies with the recommendations of the American College of Sports Medicine (ACSM) [57], with a warm-up phase, the main part, and a cooling-down part, as can be seen in Fig. 2. This distribution also allows incorporating dual tasks, games, and tasks aimed at stimulating social interaction.

To perform the exercise, a series of guidelines and progressions will be followed, which are described in the Additional file 1. The physiotherapists that will apply this PE protocol in the participating PC centres, will receive training on how to perform the program, what to say to patients, and how to resolve conflicts that may arise during the development of the program.

#### Control group intervention

The control group will receive the standard care treatment carried out in the PC physiotherapy units that is supported by the current physiotherapy protocols in primary care of Castilla y León [58]. The treatment consists of 15 sessions of thermotherapy and analgesic electrotherapy in the area of pain, and the guidelines for the exercises recommended by the SERMEF (Spanish Society of Physic Medicine and Rehabilitation) [59].

### Sample size calculation

A total of 63 individuals by each group of treatment will allow detecting, in numerical variables, differences between pairs of group mean in a magnitude corresponding to 50% of the standard deviation (an effect size of 50%) with a probability of 80%, keeping the type I error at a 5% level. By taking into account an attrition rate of 25%, it would be necessary to include 78 individuals in each group. The calculation has been done using G*Power 3.1.

### Statistical analysis

Numerical variables will be summarised with means and standard deviations and categorical ones with percentages. Those numerical variables showing a distribution very far from the normal one will be transformed for getting resulting distributions close to the normal one, or in the opposite case, they will be summarised by using percentiles. For the graphical representation of the numerical variables’ distribution in each treatment group, we will use box and whisker plots. For representing categorical variables, pie charts will be used. In order to test the equality of means for numerical variables, we will apply the Student’s t, if the corresponding distributions are not far from the normal one. In the opposite scenario, we will calculate the Wilcoxon–Mann–Whitney statistics. For testing the association between qualitative variables, we will use Chi-squared statistics, if it is allowed by the observed frequencies in the associated joint distribution. On the contrary, we will re-categorise these variables for reducing them to dichotomous ones, if necessary, and will apply the Fisher exact test. As a function of the observed response to the treatment in numerical variables or the observed change in them, we will build several definitions of success/failure for the result of treatment in individuals. We will fit logistic regression models for predicting the success of the treatment by using the previously mentioned definitions. In these models, those variables which show a statistically significant relationship with the corresponding response variables in the univariate analysis and the variable selection procedures will be included. These models will provide us, as a by-product, odds ratio values for estimating the comparative risk of success associated with each explanatory variable corrected by the presence of the other variables included in them. We will obtain estimates for the sensitivity/specificity of predictive rules derived from these models. It will be considered as statistically significant *p*-values lower than 0.05. Data analysis will be carried by using the R statistical software.

### Outcomes variables

**Fig. 3:**
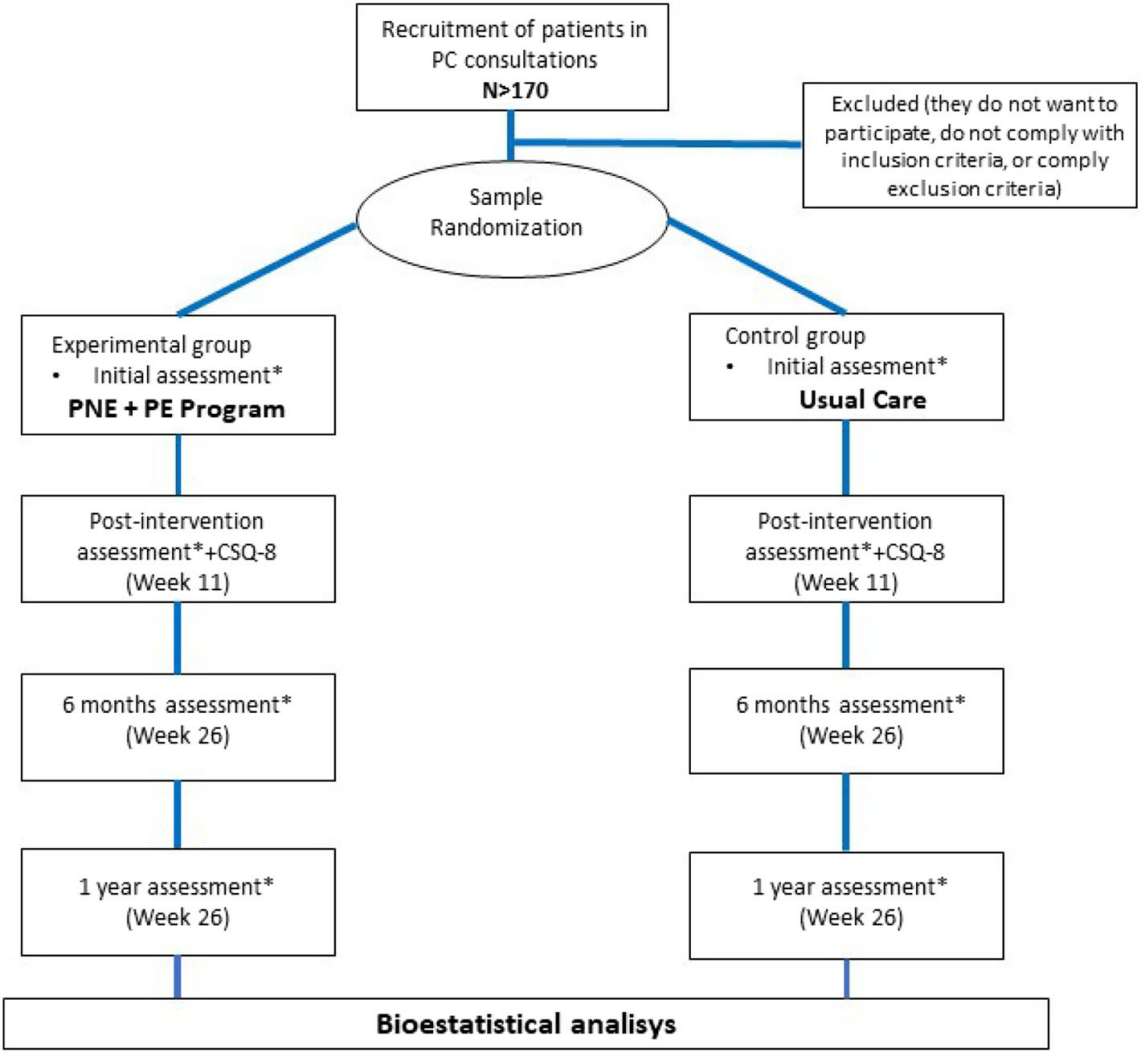
Flowchart of the study

All variables will be measured before the intervention, after the intervention (week 11), at six months, and a year. Flowchart of the study can be seen in Fig. 3.

#### Initial personal and sociodemographic variables

- Age (in years)
- Sex
- Health Centre
- Level of studies (no studies, basic education, secondary school, university studies)
- Employment situation (student, active, active in temporary disability, unemployed, domestic occupation)
- Marital status (single, widowed, couple, separated or divorced)
- Weight (kg), height, body mass Index (weight [kg]/height [m^2^]), and abdominal perimeter.
- Time since the onset of symptoms (months)
- Pain areas (McGill pain maps) [60]

#### Main outcomes variables

The primary outcome measure will be the difference between groups in health-related quality of life changes at different times. The Spanish version of the health Survey SF-36 V2 will be used [61, 62]. SF-36 is a 36-item scale built to assess health status and quality of life. It produces a profile of eight levels of functional health and wellness scores, as well as a summary of psychometric-based mental and physical health. Each scale transforms directly into a scale of 0–100. Higher scores indicate a better quality of life in general.

#### Secondary outcome variable

- Pain intensity: VAS scale (0–100 mm) [63]. Participants will be shown a horizontal line 100 mm long which ranges from 0 (“no pain”) to 100 (“the worst pain imaginable”). The participants will draw a mark on the line at a point which best represents the pain they are experiencing at the moment it is being measured. Higher scores indicate a higher pain level.
- Pressure pain threshold (kg/cm^2^). To measure this the protocol described in the study by Neziri et al. [64] will be used, measuring four points using the procedure described by Fisher [65]. The reference points will be the middle point in a horizontal line drawn between the posterior border of the acromion and the spinal process of the seventh cervical vertebra, bilaterally, and the middle point of a horizontal line drawn between the highest part of the superior border of the iliac crest and the spinal process at the same height, also bilaterally. This protocol has been validated in an asymptomatic population and can be useful when evaluating patients with central sensitisation processes [64, 66]. A Fisher digital algometer of the Wagner brand will be used. Digital algometry also presents good interobserver reliability [67, 68]. The threshold will be determined when the sensation of pressure turns to a painful sensation for the patient. Two measurements will be made at each point, increasing the pressure to a rate of 1 kg/cm^2^ per second. The average of two consecutive measurements shall be determined [66].
- Catastrophising. The Spanish version of the Pain Catastrophising scale (PCS) will be used [69]. This is a brief 13-item questionnaire that assesses pain-related behaviours and cognition. Scores range from 0 to 52, with higher scores indicating a higher level of catastrophising [70].
- Kinesiophobia: The Spanish version of the Tampa Scale of Kinesiophobia (TSK-11) will be used. This is a self-reported questionnaire containing 11 items designed to assess a patient’s fear of moving and re-injury. Scores range between 11 and 44 points, with a higher score indicating higher levels of kinesiophobia.
- Central Sensitisation: The Spanish version of the Central Sensitisation Inventory will be used [72]. This is a self-reported questionnaire used to detect the presence of central sensitisation. It contains 25 items registering the frequency of each symptom on a Likert scale from 0 (never) to 4 (always), the highest score being 100 points. Higher scores are associated with higher levels of central sensitisation. The questionnaire provides reliable and valid data that quantify the severity of various symptoms of central sensitisation [73].
- Disability: The Roland–Morris disability questionnaire will be used [74]. This is a self-reported disability questionnaire consisting of 24 questions specifically related to physical functions that may be affected by back pain. Scores range from 0 to 24 points, where a higher score indicates a higher level of disability.
- Body mass index: Changes in body mass index will be assessed. Weight values measured in kilograms and height measured in meters will be combined to report the BMI in kg/m^2^.
- Medication intake: The medication intake by types of drugs will be registered by means of a survey. The results will be expressed in weekly doses by the type of drug taken.
- Health Services visits: Visits to the family doctor, emergency services, and medical specialists will be registered during the follow-up period.
- Satisfaction with the treatment received: The Spanish version of the Client Satisfaction Questionnaire (CSQ-8) will be used [75]. The CSQ-8 is a self-report tool used to assess satisfaction with health services.

## Discussion

This study will provide new information on the effectiveness of a new program based on PNE and PE in the Spanish National Health Services, in the region of Castilla y León. The results of this study may be useful in future planning of the management of chronic pain in health services and provide the basis for future cost-effective studies, as well as optimising the available resources for more effective management of CSP.

Using a clinical trial to assess a health service is not something new. However, changes in health services affect a great number of people and require modifying structures and cultures of service. Therefore, it is mandatory to gather the information available with solid and robust evidence and test it in its context prior to modifying the services.

Previous studies suggest that management based on PNE and PE improves the quality of life of patients with CSP. None of these studies has been conducted using group work in a primary healthcare environment inside the Public Health System. The sample size used in the study may allow reaching solid conclusions about the effectiveness of the proposed treatment. Study results with an effect size superior to 0.8 (Cohen’s d) in the different variables measured could be the starting point to change the therapeutic management used in the first level of care for patients suffering from chronic musculoskeletal pain. Group work may also allow optimisation of the available resources and also allow the program to reach more patients.

Initially, our study does not include tools that allow us to quantify if the intervention produces changes on a neuroplastic and structural level and changes related to the connectivity between different brain areas. However, if the clinical results regarding improvement in the quality of life, function, and pain reduction are positive, a second study with dMRI and resting state fMRI is planned to assess whether changes occur at the brain level.

There is controversy as to whether individual or group treatments are best. In our field of care, due to the high pressure of care and in order to optimise resources, we have opted for group treatment. We take advantage of the fact that the group strategy itself is a very powerful therapeutic tool, as it extols values such as group feelings, stimulates, improves self-esteem, and generates more adherence than the individual strategy [45–47]. However, this does not prevent each participant from receiving individualised guidelines for the therapeutic program.

## Supplementary information

**Additional file 1:** PNE and PE Program Description.

## Data Availability

Not applicable for that section.

## Abbreviations

CMP: Chronic musculoskeletal pain
CS: Central sensitisation
CSP: Chronic spinal pain
PC: Primary care
PE: Physical exercise
PNE: Pain neuroscience education
RCT: Randomised clinical trial
