# Supplementary material for: Pain neuroscience education and physical exercise for patients with chronic spinal pain in primary healthcare: a randomised trial protocol
Source: BMC Musculoskelet Disord. 2019 Nov 3;20:505. doi: 10.1186/s12891-019-2889-1 (PMC6825712; doi:10.1186/s12891-019-2889-1)
Supplement: Supplementary file 1 — Additional file 1: PNE and PE Program Description. [file 12891_2019_2889_MOESM1_ESM.docx]

**Additional file 1: PNE and PE Program Description.**

This protocol follows the recommendations of the most recent clinical practice guidelines, which must be applied at the first level of care for patients with chronic back pain(27–31). Active coping strategies have shown better results than passive treatments, the latter have been the most used in the Public Health System. Given the need for a new paradigm for the treatment of pain, with a greater weight of active coping strategies that can be applied from the first level of care, the basic characteristics of the protocol developed are described below.

**1. Educational intervention. Pain Neuroscience Education**.

Educational content has been designed for patients, to be taught in six sessions. The first four sessions are 1.5 hours long and are given at a frequency of one or two weekly sessions. After completion of the second part of the therapeutic exercise program, an additional reinforcement education session is held. Three months later, the final session is given to solve doubts, and to provide additional information to the educational program.

The contents of the different sessions are the following:

**1.1. Basic contents of the Pain Neuroscience Education Program**

- Pain is an alarm system, a protection system.
- Pain is not always the same as tissue damage (pain is not always the same as nociception)
- Differences between acute and chronic pain.
- Neurophysiology of chronic musculoskeletal pain (explanation of central sensitisation, of the evaluative error, and the neuroplastic changes that contribute to pain chronification).
- Consequences of chronic pain (kinesiophobia, fear-avoidance behaviours, social isolation)
- Reversibility of the functional and structural alterations that cause persistent pain (neuroplasticity mediated by cognition and movement)
- Tools for the patient.

**Distribution of contents by session is as follows:**

| Session no. | Basic contents |
| --- | --- |
| Session 1 | - Epidemiology of chronic pain. - Pain as an alarm system. - Pain is not equal to damage. - Psychosocial and cultural aspects of pain. |
| Session 2 | - Differences between acute and chronic pain - Danger assessment system. - Amplification and inhibition systems. - Pain as a brain response. |
| Session 3 | - Consequences of chronic pain and central sensitisation. - Movement, motor control disorder, and kinesiophobia. - Fear-avoidance behaviours. - Pain catastrophising - Structural and functional disturbances that generate persistent pain. |
| Session 4 | - Knowledge as a tool in the evaluative process of sensory stimuli. - The belief system and its epigenetic effects. - Reversibility of structural and functional changes. - Neuroplasticity mediated by cognitive and somatosensory stimuli, and physical exercise. - Benefits of group exercise. |
| Session 5  *Review* | - Review of the contents covered in the first four sessions, and of the most relevant aspects of the PE group sessions. - Complementary material handout. Book for patients: “Educación en neurociencia del dolor: una nueva visión. Dossier para pacientes” (” Pain neuroscience education: a new vision. Patient handout”)(76). |
| Session 6  *End review* | - Review of basic content. - Resolution of doubts - Shared experiences. - Recruitment of “expert patients” for successive editions. |

**1.2. Type of educational intervention; Session pattern.**

The teaching staff is required to know the knowledge and beliefs the patients have about their pain, and about the cause of its perpetuation. In general, patients who attend the program will have beliefs based on a mechanical and pathoanatomical model of pain. “The greater the damage or injury to the tissues, the greater the pain will be”. Overthrowing these beliefs is one of the most difficult aims in the first stage of the program. The new knowledge brought to patients clashes head-on with the biomedical and mechanist models they have learned by various means. It is essential to dedicate at least a complete session of 1.5 hours to overthrowing these false beliefs with updating their knowledge. The same needs to be done with other limiting beliefs that may be identified. The rest of the contents of the program are well understood by the patients.

During the sessions, the patients intervene whenever they deem appropriate, or when invited to participate by the speaker. Active listening of all their doubts and comments is carried out, to which an answer is given at that moment unless they refer to contents that will be addressed further on in the program. For the explanation of some of the neurophysiological aspects, some metaphors, and graphic material will be used. The team has made sure that all of the information given to the patients is well understood. In order for this to be possible, the teaching team has had the advice of an expert in Psych pedagogy and adult education. In order to facilitate the transfer of knowledge, pedagogical resources are used to simplify the process. The therapist carrying out the educational intervention, will at all times, empathise with the patients and send positive messages(77). To this end, the times when the patients are merely receivers of information have been dosed with the times in which they actively participate. Great care has been taken with the graphic material accompanying the presentations, and infographics have been created to facilitate the process. In all the sessions, explanatory and motivational videos have been used.

Also, several pilots have been carried out to ensure that the contents provided are understood and correctly interpreted.

In order to optimise resources and taking into account that the program will be carried out in primary care centres in the public network, it was decided to conduct the educational sessions in groups. We take advantage of the fact that the group strategy itself is a very powerful therapeutic tool, as it extols values such as group feelings, stimulates, improves self-esteem, and generates more adherence than the individual strategy(45–47). The group intervention is not an obstacle to having individual reinforcement sessions with patients who require more time to cover some aspects of the educational program that may be relevant to their clinical case, and that has been detected during the initial assessment.

The educational intervention is reinforced in the second part of the program since during the exercise sessions, the therapists will be continually explaining why each of the activities is done and the physiological effects it will generate.

**1.3. Support material and recommended readings:**

**Links to web pages:**

- Retrain Pain: <https://www.retrainpain.org/>
- Sociedad Española de Fisioterapia y dolor (Spanish Society of Physiotherapy and Pain): <https://www.sefid.es/>
- Arturo Goicoechea’s blog:<https://arturogoicoechea.com/>
- Olga Sacristán’s web page:<https://olgasacristan.com/>
- NOI Group: <http://www.noigroup.com/>
- Pain in motion: <http://www.paininmotion.be/>
- Greg Lehman’s web page: <http://www.greglehman.ca/>
- Better movement: <https://www.bettermovement.org/>

**Books:**

- Migraña. Una pesadilla cerebral (Migraine. A brain nightmare)(78). A. Goicoechea. Ed. Desclée de Brouwer. 4ª ed. 2016
- Explicando el dolor (Explaining Pain)(79). D. Butler y L. Moseley. NOI Group publications. 2010. Australia
- Recovery strategies. Greg Lehman. Free book available at <http://www.greglehman.ca/>
- Therapeutic neuroscience education: Teaching people about pain(80). A. Louw y E. Puentedura. International spine and pain Institute. 2013. USA.
- Educación en neurociencia del dolor. Una nueva visión: dossier para pacientes (Pain neuroscience education: A new vision. Patient handout) (76). M.A. Galán y F. Montero. Gerencia Regional de Salud. 2018.

**2. Physical Exercise**

The second part of the program consists of 18 sessions of Physical Exercise, led by a Physiotherapist, and will be carried out during six weeks at a frequency of three weekly sessions. We know that exercise is an excellent therapeutic tool that allows us to improve function, reduce disability, and activate mechanisms of endogenous analgesia. In the case of patients with central sensitisation, prescribing exercise is a bit more complex, as initially not every type of exercise is adequate(34). We will start with simple exercises that will be gradually increased in intensity and complexity. Even though in the first sessions patients might experience an increase in pain, this will not be a sufficient reason to slow down the progression foreseen in the programme

Graded exposure to exercise decreases kinesiophobia levels and fear-avoidance behaviours. The progression made during the sessions allows for an improvement in physical qualities, function, and consequently, a decrease in disability(81). These changes are good enough reason for the patient to increase their self-esteem.

The exercises proposed have been designed to try and create neuroplastic changes and increase neurogenesis.

**2.1 Aims of the proposed physical exercise:**

- Improving function and physical condition.
- Improving representational health as a step towards correct execution of movement.
- Physical conditioning by improving basic physical abilities such as strength, speed, balance, resistance, and coordination.
- Modifying motor dysfunctional motor patterns that appear as a protective response to painful experience.
- Increasing aerobic capacity to activate endogenous mechanisms for pain control.
- Improving kinesiophobia.
- Improving fear-avoidance behaviours.
- Stimulation of social interaction and return to work (82)
- Decreasing pain intensity.

**2.2. General program guidelines:**

- The exercises will be of increasing difficulty, starting in more stable positions and simple movements, and progressing to more unstable positions and more complex movements, so they will always be a challenge to the patient.
- As the program progresses, with the consequent improvement in the execution of the exercises, **dual task** work activities will be incorporated (simultaneous physical and cognitive work). Double task activities will increase as the program progresses.
- The main part of the session that includes moderate aerobic exercise will be gradually increased and will be accompanied by a dual task activity.
- All sessions will incorporate a **ludic part** during the main part of the session. Popular and made-up games will be included, which help improve neuroplasticity. Games are a very useful tool in these patients, as it serves as a distraction strategy that is of great help in overcoming kinesiophobia and in increasing exercise load without increasing fatigue perception. Through play, aerobic capacity is improved, and the interaction and cohesion of the group are favoured.
- The cooling down part of the session will always include, apart from stretching and flexibility exercises, a short practice of relaxation exercises and/or mindfulness exercises. This way of ending the session allows for the patient to improve their ability to modify their attentional focus, which is very beneficial for those patients who have excessive hypervigilance.
- In all the sessions, one of the purposes is to encourage creativity through games and activities for the patients to do at home, such as coordination and skill exercises carried out with both hands, writing with the non-dominant hand, drawings made with both hands using symmetrical and asymmetrical scribbling, or preparation of cooking recipes that they have never made before.
- In all the sessions there will be a challenge for the members of the group that requires motor training, so they show the rest of the participants in the following session that they have been able to do it.
- Patients receive positive reinforcement constantly, both verbal and non-verbal (applause, body language...) from the physiotherapist and from the other participants.
- Excessive corrections in physical activity are continually avoided. Being aware that motor performance can be improved, gradual exposure and reduction of kinesiophobia will allow a better execution of all those functional movements necessary for daily life.
- Through the game, the physical contact established by the components of the group increases until the embrace becomes an important part of the PE sessions(83).
